# 髓系肿瘤合并噬血细胞性淋巴组织细胞增多症8例临床特征

**DOI:** 10.3760/cma.j.issn.0253-2727.2023.11.012

**Published:** 2023-11

**Authors:** 艳旗 李, 宇卉 张, 广帅 滕, 耐博 胡, 艳 王, 依璠 段, 洁 白

**Affiliations:** 天津医科大学第二医院血液科，天津 300211 Department of Hematology, Second Hospital of Tianjin Medical University, Tianjin 300211, China

噬血细胞性淋巴组织细胞增多症（HLH）是一种罕见的危及生命的综合征，根据发病原因可分为原发性或继发性[1]。恶性肿瘤特别是血液系统恶性肿瘤是成人继发性HLH发病最常见的原因（约占45％）[2]，淋巴瘤相关的HLH已有大量研究[3]，但髓系肿瘤相关的HLH报道有限。本研究回顾性分析了8例髓系肿瘤合并HLH临床特征、遗传学与表观遗传学基因突变情况以及疾病转归，现报道如下。

## 病例与方法

1. 病例资料：本研究为回顾性研究，收集2017年11月至2022年5月天津医科大学第二医院血液科收治的8例髓系肿瘤合并HLH患者的临床和实验室检查资料，诊断均符合HLH-2004标准和2016 WHO髓系肿瘤诊断指南[4]。

2. 细胞因子检测：采用AimPlex多因子流式检测技术检测血清样本中各细胞因子的表达。应用IFN-γ/IL-1β/IL-2/IL-4/IL-5/IL-6/IL-8/IL-10/IL-12p70/IL-17A/IL-17F/IL-22/TNFα/TNFβ检测试剂盒（北京旷博生物技术股份有限公司），通过Canto plus流式细胞仪（美国BD公司）进行检测。

3. 染色体核型分析：染色体标本取自骨髓，肝素抗凝。经常规24 h短期培养，收获后制片，R+G显带。核型描述依据《人类细胞遗传学国际命名体制（ISCN2013）》。

4. 二代测序（NGS）：取患者骨髓标本提取DNA，对362个血液肿瘤相关基因目标区域进行富集扩增，并采用超高多重RCR外显子富集技术进行高通量基因测序，平均测序深度为800×；使用lon Reporter系统和Variant Reporter软件进行突变分析；利用dbSNP、1000 Genomes、Polyphen-2和COSMIC等数据库进行变异注释及氨基酸突变分析。

5. 全外显子测序（WES）：构建插入片段为200 bp的文库，采用高效液相探针捕获平台捕获目标序列，利用Illumina HiSeq X二代测序平台，对文库进行双末端（Paired-end，PE）测序，测序读长为2×150 bp。测序数据以GRCh38.p13为参考基因组，结合dbSNP、COSMIC、ClinVar等多个公共数据库和荻硕贝肯自有数据库对数据进行整理、编辑和生物信息分析。

6. 统计学处理：对所收集的数据、治疗方法及预后转归进行回顾性综合分析，连续变量用中位数（范围）表示，分类变量用例数（构成比）表示。总生存（OS）时间定义为髓系肿瘤患者确诊HLH至任何原因死亡的时间，采用Kaplan-Meier法进行生存分析。

## 结果

1. 临床特征：8例患者中，其中急性髓系白血病（AML）5例，骨髓增生异常综合征（MDS）1例，原发性血小板增多症继发骨髓纤维化（Post-ET MF）1例，慢性粒-单核细胞白血病（CMML）1例，男5例，女3例，中位年龄61.5（34～79）岁。5例HLH发生于疾病的初始或疾病进展阶段，考虑为肿瘤诱发的HLH（M-HLH），3例发生于化疗后骨髓抑制期，称为化疗期间发生的HLH（Ch-HLH）。其中合并感染5例（62.5％），均为肺感染，1例合并EB病毒感染（表1）。8例患者均有持续性发热（体温38～40°C）（100.0％），脾大5例（62.5％），所有患者均有不同程度血细胞减少（100.0％），其中三系减少5例（62.5％），两系减少3例（37.5％）。血清铁蛋白水平均升高（100.0％）；甘油三酯升高2例（25.0％）；纤维蛋白原降低7例（87.5％）；骨髓吞噬现象2例（25.0％）；乳酸脱氢酶水平升高 8例（100.0％）；肝功能异常7例（87.5％），包括转氨酶、胆红素升高；C反应蛋白水平升高8例（100.0％）。

**表1:** 髓系肿瘤合并HLH基本情况、治疗及确诊HLH后生存时间

例号	肿瘤类型	感染	EB病毒感染	发生HLH时临床阶段	发生HLH时染色体核型	HLH治疗	HLH确诊后生存时间	基因突变情况
1	CMML	肺	无	初始阶段	46,XY[20]	芦可替尼	11 d	U2AF1 Exon2（Ⅰ类，46.4%）、EZH2 Exon16（Ⅰ类，43.5%）、XPO1 Exon14（Ⅱ类，42.7%）
2	MDS、食管癌	肺	无	初始阶段	未检测	激素	92 d	TP53 Exon5（Ⅰ类，71.5%）
3	AML-M_2b_	无	无	初始阶段	45,X,−Y,t(8;21)(q22;q22)[14]/46,XY[6]	激素	214 d	KDM6A Intron18（Ⅱ类，12.5%）、ZBTB7A Intron2（Ⅱ类，45.1%）
4	Post-ET MF	肺	有	初始阶段	46,XY[20]	芦可替尼	23 d	NRAS Exon2（Ⅰ类，12%）、MPL Exon10（Ⅰ类，51.46%）、ASXL1 Exon12（Ⅰ类，3.06%）、ETV6 Exon4（Ⅱ类，15.91%）
5	AML-M_5_	肺	无	化疗期间	47,XY,+9[4]/47,XY,+21[3]/46,XY[13]	支持治疗	14 d	ARID1A Exon1（Ⅱ类，52.78%）、POT1 Exon13（Ⅱ类，50%）、NOTCH1 Exon34（Ⅱ类，53.85%）、KMT2D Exon31（Ⅱ类，45.28%）、STAT5B Exon19（Ⅱ类，34.86%）
6	AML-M_5_（MDS转）	无	无	化疗期间	dup(1),20q−,+8	支持治疗	9 d	U2AF1 Exon2（Ⅰ类，42.2%）、KRAS Exon2（Ⅰ类，16.8%）、EP300 Exon10（Ⅱ类，31.3%）
7	AML（MDS转）	无	无	疾病复发	der(3),der(5;17),der(7),−8,der(9),−12,−13,−15,−16,add(19q),+mar1,+mar2,+mar3	依托泊苷+地塞米松	8 d	TP53 Exon5（Ⅱ类，41.73%）
8	AML-M_5_（MDS转）	肺	无	化疗期间	+2,+3,+der(4),−5,+6,−7,add(8p),+9,+10,+11,+12,−13,add(16q),+17,+18,−19,add(19q)×2,+20,+ 21,+22	依托泊苷+地塞米松	19 d	TP53 Exon7（Ⅰ类，78.85%）

注 HLH：噬血细胞性淋巴组织细胞增多症；CMML：慢性粒-单核细胞白血病：MDS：骨髓增生异常综合征；AML：急性髓系白血病；Post-ET MF：原发性血小板增多症继发骨髓纤维化；Ⅰ类：与疾病密切相关的热点突变；Ⅱ类：与疾病可能相关的突变

2. 实验室检查资料结果：3例髓系肿瘤合并HLH患者进行了细胞因子检测，IL-6［（82.554±54.144）µg/L］、IL-8［（86.933±43.650）µg/L］、IL-17A［（14.727±6.453）µg/L］等细胞因子水平均明显升高。染色体分析结果显示5例患者有不同程度的染色体核型异常，例7是AML-M_5_（MDS转）患者，发生HLH时染色体核型由最初−Y,der（5;17）,−7,del（7）,−10,−16,add（19）演变为der（3）,der（5;17）,der（7）,−8,der（9）,−12,−13,−15,−16,add（19q）,+mar1,+mar2,+mar3，出现了13种附加异常，染色体复杂性增加（表1）。8例患者进行了NGS检测，其中3例TP53突变，2例U2AF1突变。存在DNA修复相关基因（TP53）、转录因子相关基因（ETV6等）、RNA剪接相关基因（U2AF1等）、组蛋白修饰相关基因（EZH2、ASXL1等）、信号通路异常相关基因（MPL、NRAS等）突变的患者分别占37.5％、12.5％、25％、25％和12.5％。6例患者进行WES检测，均未检测到先天性噬血相关基因突变。此外，分析本中心169例髓系肿瘤不合并HLH患者WES结果，发现5例患者［3例原发性骨髓纤维化（PMF）、1例MDS、1例真性红细胞增多症］存在先天性噬血相关基因，其中4例为杂合突变（UNC13D突变3例，STX11突变1例），1例存在SH2D1A:p.A3S纯合错义点突变。

3. HLH的治疗及预后转归：单纯激素治疗2例，芦可替尼治疗2例，依托泊苷+地塞米松治疗2例，支持治疗2例。8例患者均已死亡，生存分析显示确诊HLH后中位OS时间14（95％*CI* 3～25）d。M-HLH与Ch-HLH两组患者确诊HLH后中位OS时间分别为23（95％*CI* 0～48.8）d和14（95％*CI* 6～22）d。

## 讨论

M-HLH与Ch-HLH应分别看待，化疗后病毒、真菌和细菌感染可能是Ch-HLH的主要诱因[5]，髓系肿瘤包括骨髓增殖性肿瘤（MPN）、MDS及AML。基于不同髓系肿瘤在遗传学和表观遗传学相关基因突变有较多的重叠，本研究综合分析其合并HLH的临床特征及预后。

Delavigne等[6]总结了343例接受化疗的AML患者，32例（10％）AML患者化疗期间合并HLH，其中24 例（75％）有微生物学证据，合并HLH的AML患者中位OS时间为14.9个月，明显短于无HLH的AML患者（22.1个月）。Lackner等[7]报道AML中M_5_型更容易发生HLH（占所有AML的30％），他们认为这种急性单核细胞白血病亚型对HLH的发展具有易感性，可能是由于单核细胞白血病细胞自身释放的细胞因子和噬血单核细胞过度激活所致。本研究5例合并HLH的AML患者中3例为M_5_型，经包括病原学NGS检测等多种方法证实无任何感染证据，OS时间分别为14、9、19 d。单核细胞白血病易发HLH的机制仍需要进一步深入研究。尽早识别高风险HLH的患者，积极治疗AML原发病，是提高AML合并HLH患者生存率的重要举措。

Sun等[8]发现1例MDS继发HLH患者存在DNMT3A p.Arg736His（c.2207G>A）和DNMT3A p.Leu859Ter（c.2576T>A）双等位基因突变，推测DNMT3A突变引起的表观遗传学异常导致与免疫失调相关的异常干细胞基因表达，从而增加了免疫失调和炎症反应，诱发HLH。包括TET2、ASXL1和DNMT3A在内的髓系肿瘤特异性表观遗传学基因突变可诱导炎症小体激活[9]，并加剧炎症反应[10]，可能与AML患者HLH发生相关[8]。与国外学者的研究结果不同，本研究8例患者中，仅1例检出ASXL1基因突变，未检测到TET2及DNMT3A基因突变；而3例（37.5％）患者检测出TP53基因突变。在另一项非霍奇金淋巴瘤诱发HLH研究中，约21.24％的患者伴随TP53突变[11]。不同体细胞突变影响细胞DNA修复、转录因子、RNA剪接、表观遗传学修饰、DNA甲基化等过程[12]。本研究我们发现，8例合并HLH的髓系肿瘤患者中3例TP53异常，提示DNA修复异常可能与HLH有一定的相关性。

遗传学不稳定性是细胞中染色体数量和（或）结构异常的状态[13]。髓系肿瘤合并HLH患者更容易合并核型异常，如+8染色体异常[8]。另有报道1例AML合并HLH患者伴有复杂核型t（8;21;22）[14]，Farias等[15]报道1例AML患者化疗前染色体为46,XX，化疗后患者发生HLH时染色体核型为1p,2p,3p,6q,8p,10qter,16q,17q，提示遗传学的不稳定也可能会诱发HLH[15]。本研究8例髓系肿瘤患者中，5例存在核型异常，其中1例AML（MDS转）患者出现染色体核型演变，提示对于细胞遗传学不稳定的髓系肿瘤患者，应警惕HLH的发生。

原发性HLH主要是由于基因缺陷（如PRF1、UNC13D等基因突变）导致细胞毒性T细胞和NK细胞释放穿孔素及颗粒酶等功能受损，从而不能有效杀伤靶细胞，引发过度免疫激活，其主要发生于儿童，多有明确的HLH家族史[1]。本研究8例合并HLH的髓系肿瘤患者中6例进行WES检测，均未发现噬血相关基因突变，暂不考虑先天性因素导致的HLH。我中心169例进行WES的髓系肿瘤患者中，5例发现了HLH相关基因突变，其中4例为杂合突变，1例PMF患者存在SH2D1A:p.A3S纯合错义点突变。但是此5例患者并没有发生HLH。而因此，噬血相关基因突变在髓系肿瘤发生HLH的风险意义有待进一步验证。

总之，髓系肿瘤相关HLH预后极差，遗传学的不稳定、TP53等基因突变与髓系肿瘤并发HLH相关性有待进一步研究。噬血相关基因突变在髓系肿瘤患者中发生HLH的风险预测需要更多探索。
